# Fluorofenidone inhibits epithelial-mesenchymal transition in human lens epithelial cell line FHL 124: a promising therapeutic strategy against posterior capsular opacification

**DOI:** 10.5935/0004-2749.20210040

**Published:** 2021

**Authors:** Hua Zhuang, Ning-Xuan Zheng, Lin Lin, Wu-Zhen Zhang, Wan-Yu Zhang, Qin-Qi Yu, Wei Xu

**Affiliations:** 1 Fuzhou Aier eye Hospital, Fuzhou, China; 2 Aier School of Ophthalmology, Central South University, Changsha, Hunan Province, China; 3 Fujian Center for Disease Control and Prevention, Fu Zhou, Fujian Province, China; 4 Women and Children’s Hospital Affiliated to Xiamen University, Xiamen, Fujian Province, China; 5 Xianyou maternal and Child Health Hospital, Putian, 351200, Fujian Province, China; 6 Fujian Children’s Hospital, Fu Zhou, Fujian Province, China; 7 Yongzhou First People’s Hospital, Hunan Province, China; 8 The 1st affiliated hospital of Fujian Medical University, Fu Zhou, Fujian Province, China

**Keywords:** Transforming growth factor beta2, Fluorofenidone, Lens, Cataract, Infant, Fator de crescimento transformador beta2, Fluorofenidona, Lentes, Catarata, Lactente

## Abstract

**Purpose:**

The present study aimed to investigate the inhibitory effect of fluorofenidone against transforming growth factor β2-induced proliferation and epithelial-mesenchymal transition in human lens epithelial cell line FHL 124 and its potential mechanism.

**Methods:**

We evaluated the effect of fluorofenidone on proliferation and epithelial-mesenchymal transition of human lens epithelial cell line FHL 124 in vitro. After treatment with 0, 0.1, 0.2, 0.4, 0.6, and 1.0 mg/mL fluorofenidone, cell proliferation was measured via MTT assay. Cell viability was evaluated by lactate dehydrogenase activity from damaged cells. FHL 124 cells were treated with different transforming growth factor β2 concentrations (0-10 ng/mL) for 24 h and the expression of CTGF, α-SMA, COL-I, E-cadherin, and Fn were detected via quantitative polymerase chain reaction and Western blot analysis. After treatment with 0, 0.2, and 0.4 mg/ml fluorofenidone, the expressions of transforming growth factor β2 and SMADs were detected with real-time polymerase chain reaction and Western blot analysis. Expressions of CTGF, α-SMA, COL-I, and Fn were analyzed by immunocytochemistry assay.

**Results:**

The viability of FHL 124 cells was not inhibited when the fluorofenidone concentration was ≤0.4 mg/mL after the 24h treatment. Cytotoxicity was not detected via lactate dehydrogenase assay after the 24h and 36h treatment with 0.2 and 0.4 mg/mL fluorofenidone. Transforming growth factor β2 increased mRNA and protein expression of CTGF, α-SMA, COL-I, and Fn. However, fluorofenidone significantly suppressed expression of SMADs, CTGF, α-SMA, COL-I, and Fn in the absence or presence of transforming growth factor β2 stimulation.

**Conclusions:**

Fluorofenidone significantly inhibited expression of SMADs, CTGF, α-SMA, COL-I, and Fn in FHL 124 cells. Due to noncompliance in infants, fluorofenidone may become a novel therapeutic drug against posterior capsular opacification in infants.

## Abstract (pt)

**Objetivo:**

Investigar o efeito inibidor da fluorofenidona contra a proliferação e a transição epitélio-mesenquimal induzida pelo fator de transformação do crescimento β2 na linha HLEC FHL 124 e seu mecanismo potencial.

**Métodos:**

Avaliou-se o efeito *in vitro* da fluorofenidona na proliferação e na transição epitelial-mesenquimal da linha FHL 124 de cé lulas epiteliais do cristalino humano. Após tratamento com fluorofenidona nas concentrações de 0, 0,1, 0,2, 0,4, 0,6 e 1,0 mg/mL, a proliferação celular foi medida através de um ensaio de MTT. A viabilidade celular foi avaliada pela atividade da lactato-desidrogenase liberada por células danificadas. As células FHL 124 foram tratadas com diferentes concentrações do fator de transformação do crescimento β2 (0-10 ng/mL) por 24 horas e a expressão de CTGF, α-SMA, COL-I, E-caderina e Fn foram avaliadas por qPCR e *Western blot*. Após tratamento com 0, 0,2 e 0,4 mg/mL de fluorofenidona, as expressões do fator de transformação do crescimento β2 e de SMADs foram detectadas com PCR em tempo real e *Western blot*. As expressões do CTGF, α-SMA, COL-I e Fn foram analisadas através de um ensaio imunocitoquímico.

**Resultados:**

A viabilidade das células FHL 124 não foi inibida com concentrações de fluorofenidona menores ou iguais a 0,4 mg/mL após 24 horas de tratamento. Não foi detectada nenhuma citotoxicidade pelo ensaio da lactato-desidrogenase após 24 e 36 horas de tratamento com 0,2 e 0,4 mg/mL de fluorofenidona. O fator de transformação do crescimento β2 aumentou a expressão de mRNA e proteína do CTGF, α-SMA, COL-I e Fn. Porém, a fluorofenidona suprimiu significativamente a expressão de SMADs, CTGF, α-SMA, COL-I e Fn, tanto na ausência quanto na presença de estimulação pelo fator de transformação do crescimento β2.

**Conclusões:**

A fluorofenidona inibiu significativamente a expressão de SMADs, CTGF, α-SMA, COL-I e Fn em células FHL 124. Devido à ausência de incompatibilidade em lactentes, a fluorofenidona pode vir a se tornar um novo medicamento contra a opacificação capsular posterior em lactentes.

## INTRODUCTION

Posterior capsular opacification (PCO) frequently develops after extracapsular cataract extraction or phacoemulsification surgery, which significantly compromises visual outcomes. Furthermore, the postoperative recurrent rate of PCO in infants is 100%. The existing pharmacological treatments are unsatisfactory and have toxic side effects. PCO manifests Elschnig pearl, peripheral Soemmering’s ring (central PCO in the visual axis), cortex proliferation, and cholesterol crystal in some cases. It responds to neodymium-doped *yttrium aluminium garnet* (YAG) laser capsulotomy quite well, and vision can be restored effectively and permanently in adults. However, in infants, laser capsulotomy is accompanied by unexpected vision-related complications, such damage to intraocular lens, which is much more common in infants due to their noncompliance and vigorous proliferation of human lens epithelial cells (HLECs). Even if the posterior capsule is perfectly removed, it is also much more likely for residual lens cells to migrate and proliferate into the vitreous cavity in children than in adults. As a consequence, retinal detachment may occur, which is definitely detrimental to children’s visual health, and additional vitrectomy to eliminate vitreous lens cells and repair the retina increases the risks in patients. Briefly, in infant PCO, drug conservative therapy is much safer and need to be developed than laser treatment and surgery.

It is widely known that the lens epithelial-mesenchymal transition (EMT) and migration from equator of anterior capsular to the center of posterior capsule are the common cytological bases of PCO^(1)^. EMT and collagen deposition are also the pathological processes in PCO. EMT is characterized by decreased expression of E-cadherin and increased expression of α-SMA, and α-SMA is an important sign of EMT and extracellular matrix (ECM) synthesis in HLECs^(2)^. Both TGF-β and CTGF play crucial roles in ECM synthesis and tissue fibrosis by combining with their respective receptors and promoting cell differentiation and ECM^(3)^. Moreover, fibrosis and transdifferentiation of intraocular LECs, trabecular meshwork cells, and retinal pigment epithelial (RPE) cells induced by transforming growth factor β2 (TGF-β) and connective tissue growth factor (CTGF) have been considered pathological processes for many eye diseases^(4)^. They also enhance cell proliferation, differentiation, adhesion, and other important physiological activities^(5)^. Additionally, both TGF-β and CTGF induce EMT, α-SMA, Fn, COL-I, and collagen type IV but inhibit E-cadherin expression^(6)^.

Recently, a very promising drug, fluorofenidone, also known as AKF-PD, is identified as an antiproliferative agent^(7)^. AKF-PD is a pyridyl ketone compound with a broad spectrum of antifibrosis activities^(8,9)^. Moreover, previous studies have revealed its utility in inhibition of mouse renal fibrosis caused by diabetes and unilateral ureter obstruction^(8)^. AKF-PD delivers satisfactory results in the treatment of renal interstitial fibrosis in preclinical research^(10)^. This drug has been demonstrated to possess multi-organ antifibrotic activities, such as in the kidney^(11)^, lung^(12)^, and liver^(13)^. In the field of ophthalmology, the effect of AKF-PD on the HLECs remains unexplored till now. This study aimed to determine the therapeutic effects of AKF-PD in PCO and explore the related molecular mechanism in vitro.

## METHODS

### Culture and treatment of HLEC line

HLEC line FHL 124 was provided by ATCC (Manassas, VA, USA). The cells in this study have 99.5% homology with native human lens epithelium^(14)^. They were cultivated in culture dishes with DMEM containing 5% fetal bovine serum (FBS). The cells were synchronized by replacing the nutrient medium with serum-free DMEM and cultured for 24 h when the cells had 75% confluence.

### Cell viability assay

The cell viability test was performed using two different methods: MTT assay and lactate dehydrogenase (LDH) activity.

The cell viability of lens cells in different groups was determined via MTT assay (Beyotime Company, Shanghai, China) to evaluate the effect on cell proliferation. Lens cells were cultivated in DMEM medium with 0 (control), 0.1, 0.2, 0.4, 0.6, or 1 mg/mL AKF-PD (Beyotime Company, Shanghai, China) for 4, 8, 12, 24, or 36 h, respectively. The mixture was seeded into 96-well plates at a density of 5000 cells/well. The cells were washed twice with PBS (HyClone, USA). A total of 25 µL of MTT (50 mg/mL) was added to each well, and the cells were incubated at 37°C for 4 h. Then, the culture medium of each well was replaced with 150 µL of dimethyl sulfoxide (Sigma) and shaken for 15 min. The absorbance was measured at 490 nm by a microplate reader (Thermo).

LDH is a glycolytic enzyme involved in pyruvate to lactic acid metabolism, which is present in almost all tissues or cytoplasm in the body. When the cell membrane is damaged, LDH is rapidly released. The detection of LDH was performed following the methods of Deng et al.^(15)^.

In LDH assay, LDH activity released from the damaged cells was measured after treatment with 0 (control), 0.2 mg/mL, and 0.4 mg/mL AKF-PD. The degree of cell damage was determined by detecting LDH activity in cell culture supernatant using a commercial LDH kit (Roche, Mannheim, Germany). Cell culture medium was processed, and the optical density (OD) was measured using a microplate reader at the wavelength of 450 nm. Cytolysis percentage (% cytotoxicity ) was calculated: exp. (value - low control):(high control - low control).

### Effect of TGF-_β_**2 on CTGF,**_α_-SMA, COL-I, E-cadherin, and Fn in lens cells

#### Quantitative real-time PCR (qPCR)

The cells were treated with TGF-β2 at concentrations of 0 (control group), 1 ng/mL, 5 ng/mL, or 10 ng/mL for 24h. Total cell RNAs were extracted using a TRIzol total RNA extraction kit (Invitrogen Company, Shanghai, China) following the manufacturer’s instructions. Then, reverse transcription was performed using cDNA synthesis kit from Fermentas Co., Ltd. (Lithuania). The primer pairs used are presented in table 1. qPCR was performed on Bio-Rad IQ5 thermal cycler (Bio-Rad, California, USA). The results were analyzed with BioQ software to obtain Ct value for each PCR, and the △△Ct method was used to quantify the levels of gene expression.

**Table 1:** Human primer sequences used for real-time PCR

Gene	Forward primer	Reverse primer
TGFβ2	GAGGGATCTAGGGTGGAAATGG	AGGACCCTGCTGTGCT GAGT
α-SMA	GACAATGGCTCTGGGCT	CTGTGCTTCGTCACC
	CTGTAA	CACGTA
Fn	CAGGATCACTTACGGAGAAACAG	GCCAGTGACAGCAT ACACAGTG
Col	TCTAGACATGTTCAGCTTTGTGG	TCTGTACGCAGGTG
	AC	ATTGGTG
CTGF	CTTGCGAAGCTGACCTGGAA	TCTGTACGCAGGTG ATTGGTG
E-cadherin	GAGTGCCAACTGGACCATTCAGTA	AGTCACCCACCTCT AAGGCCATC
β-actin	TGGCACCCAGCACAATGAA	CTAAGTCATAGTCC GCCTAGAAGCA
SMAD3	TCGAGCCCCAGAGCAATATT	CGTCCATGCTGTGGTTC ATC
SMAD4	ACATTGGATGGGAGGCTTCA	GATCAGGCCACCTCCA GAGA

### Western blot analysis

Lens cells were treated with TGF-β2 at concentrations of 0 (control group), 0.5 ng/mL, 1 ng/mL, 5 ng/mL, or 10 ng/mL for 24 h. The monolayer cultures were collected with cell scrapers and then lysed with 100 µL of cell lysis buffer on ice for 30 min. The cell lysates were centrifuged, and supernatants were collected. Total protein was prepared from each group. The protein concentrations in the supernatants were aliquoted and kept using the BCA method (Biocolor, Shanghai, China) for further experiments. A total of 50 µg protein per sample was electrophoresed by 10% polyacrylamide gel electrophoresis and transferred to nitrocellulose membrane (Millipore, Billerica, MA). It was blocked with 5% skimmed milk for 1 h at room temperature and incubated overnight at 4^o^C with mouse monoclonal antibodies specific to CTGF (Millipore), α-SMA (Millipore, USA), Fn (Abcam, UK), Col-1 (Proteintech, USA), and E-cadherin (Millipore) at 4°C. After washing, the membrane was incubated with secondary antibodies (anti-mouse antibody conjugated, Abcam, Cambridge, UK). The membrane was immersed in enhanced chemiluminescence solution and then exposed to an X-ray film. After hybridization of secondary antibodies, the resulting images were analyzed with ChemiImager 4000 (Alpha Innotech Corporation, California, USA).

### Inhibitory effect of AKF-PD on FHL 124 cells

#### Inhibitory effect of AKF-PD on TGF-_β_2, SMAD3, and SMAD4 in lens cells

The cells were treated with AKF-PD at concentrations of 0 (control group), 0.2, or 0.4 mg/mL for 24h.

#### qPCR

qPCR were performed as mentioned above. The primer pairs that we used were presented in table 1.

#### Western blot analysis

Western blot analysis was performed as mentioned above. The nitrocellulose membrane was treated with primary antibodies (Abcam, Cambridge) (1:1000 antiTGF-β2, 1:500 anti-SMADs) and then secondary antibodies (anti-mouse antibody conjugated, Abcam).

### Inhibitory effect of AKF-PD on TGF-_β_ 2-induced SMAD3 and SMAD4 expression in lens cells

Lens cells were treated with AKF-PD at concentrations of 0 (control group), 0.2, or 0.4 mg/mL in the presence of 10 ng/mL TGF-β2 for 24 h. qPCR and Western blot analysis were performed as mentioned above.

### The morphology observation and immunocytochemistry and grouping method were conducted as follows:

Control group; (2) 0.4 mg/mL AKF-PD for 24h; (3) 10 ng/mL TGF-β2 for 24 h; (4) co-treatment with 0.4 mg/mL AKF-PD and 10 ng/mL TGF-β2 for 24 h.

### Morphology of lens cells in vitro

Lens cells were seeded into culture dishes with DMEM containing 5% FBS. The HLECs were synchronized by replacing the nutrient medium with serum-free DMEM and cultured for 24 h when the cells had 75% confluence.

The morphology changes were observed after the treatment.

### Immunocytochemistry

FHL 124 cells were cultivated at a density of 6×104 cells/mL. The cells were fixed with 4% paraformaldehyde for 16 min. Then, the fixed cells were treated with 0.1% Triton X-100 for 10 min. The cells were subsequently incubated in 3% H2O2 for 10 min. The cells were blocked in 5% goat serum for 20 min and then incubated with mouse anti-human COL-I, Fn, CTGF, and α-SMA (1:100 dilution) for one night. Following three washes with PBS, the cells were treated with secondary antibody (polymer helper and polyperoxidase-anti-mouse IgG) for 30 min at 37^o^C. The cells were treated with DAB reagent box (ZSGB-BIO Company, Beijing, China). FHL 124 cells were stained with hematoxylin for 20 s. The slides were embedded in neutral balsam. The slides were observed through a microscope. Representative images were captured with the incorporated digital camera (Olympus image analysis system, Japan). The average positive OD was determined and analyzed by image analysis system.

### Image acquisition and statistical analysis

SPSS 13.0 statistical software was employed to conduct all statistical analyses. After treatment of lens cells with different TGF-β2 and AKF-PD concentrations, the overall comparison of protein and mRNA expressions with control group was analyzed using one-way analysis of variance, while the difference between groups was compared using Tukey honestly significant difference test. Differences with p-value <0.05 were considered statistically significant.

## RESULTS

### FHL 124 cell proliferation inhibited by AKF-PD was detected via MTT assay and LDH activity (Figure 1)

In MTT assay, there was no statistically significant difference in the control, 0.1 mg/mL, 0.2 mg/mL, and 0.4 mg/mL groups at all time points. However, cell proliferation was inhibited when AKF-PD concentration was ≥0.6 mg/mL compared with the control group (*p <0.05; **p<0.01) at all time points. The effect was more remarkable in the 1.0 mg/mL group at 36 h (**p<0.01). From the discussion above, the following studies were performed with 0.2 and 0.4 mg/mL AKF-PD after the 24h treatment.

**Figure 1:**
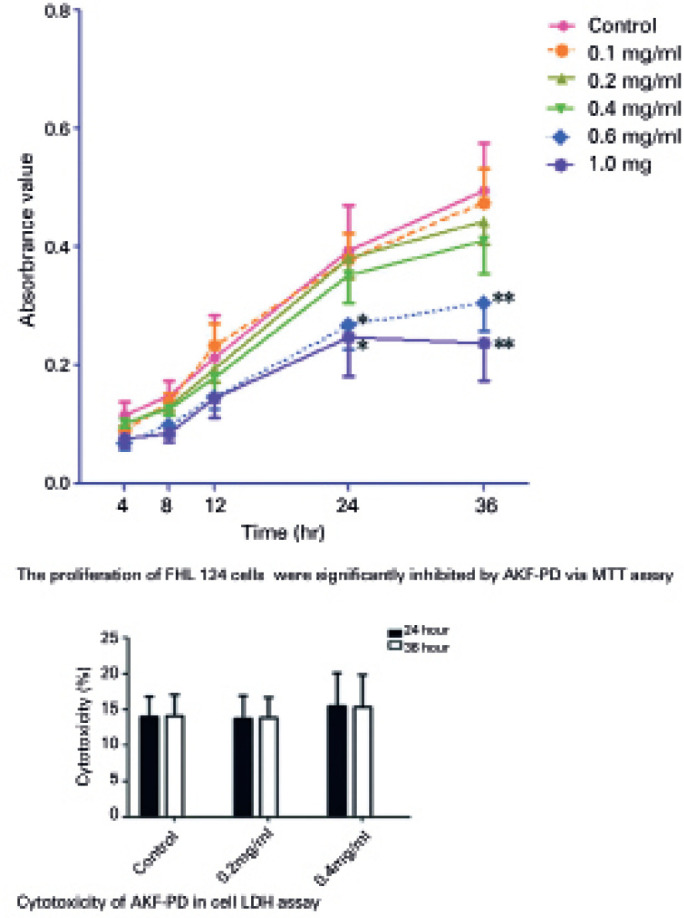
The proliferation of FHL 124 cells were detected via MTT and LDH assay.

In MTT assay, cell proliferation was inhibited when AKF-PD concentration was _≥_0.6 mg/mL compared with the control group. In LDH assay, after the 24-or 36-h treatment with AKF-PD (0, 0.2, and 0.4 mg/mL), no statistically significant difference was found between the percentage of cytolysis in AKF-PD-treated groups or control group (p>0.05). *p<0.05 and **p<0.01 were obtained comparisons between treatment and control groups at different time points.

In LDH assay, after treated with AKF-PD for 24h, the cytolysis percentages were 13.90 ± 2.9, 13.74 ± 3.2, and 15.41 ± 4.7 for the control, 0.2, and 0.4 mg/mL groups, respectively, and the cytolysis percentages after 36h were 14.10 ± 3.0, 13.84 ± 2.9, and 15.27 ± 4.5 for the control, 0.2, and 0.4 mg/mL AKF-PD groups. There was also no significant difference among the groups at 24 and 36 h, respectively (p>0.05).

### Effect of TGF-_β_**2 on the mRNA and protein expression of CTGF,**_α_-SMA, COL-I, E-cadherin, and Fn in lens cells (Figure 2)

It is confirmed in our experiment that, after the 24-h treatment of lens cells with TGF-β2, mRNA (*p<0.05; **p<0.01 compared with the control group) and protein levels (*p<0.05; **p<0.01) of CTGF, α-SMA, COL-I, and Fn were dose dependently upregulated. However, E-cadherin expression was remarkably inhibited in a dose-dependent manner.

**Figure 2:**
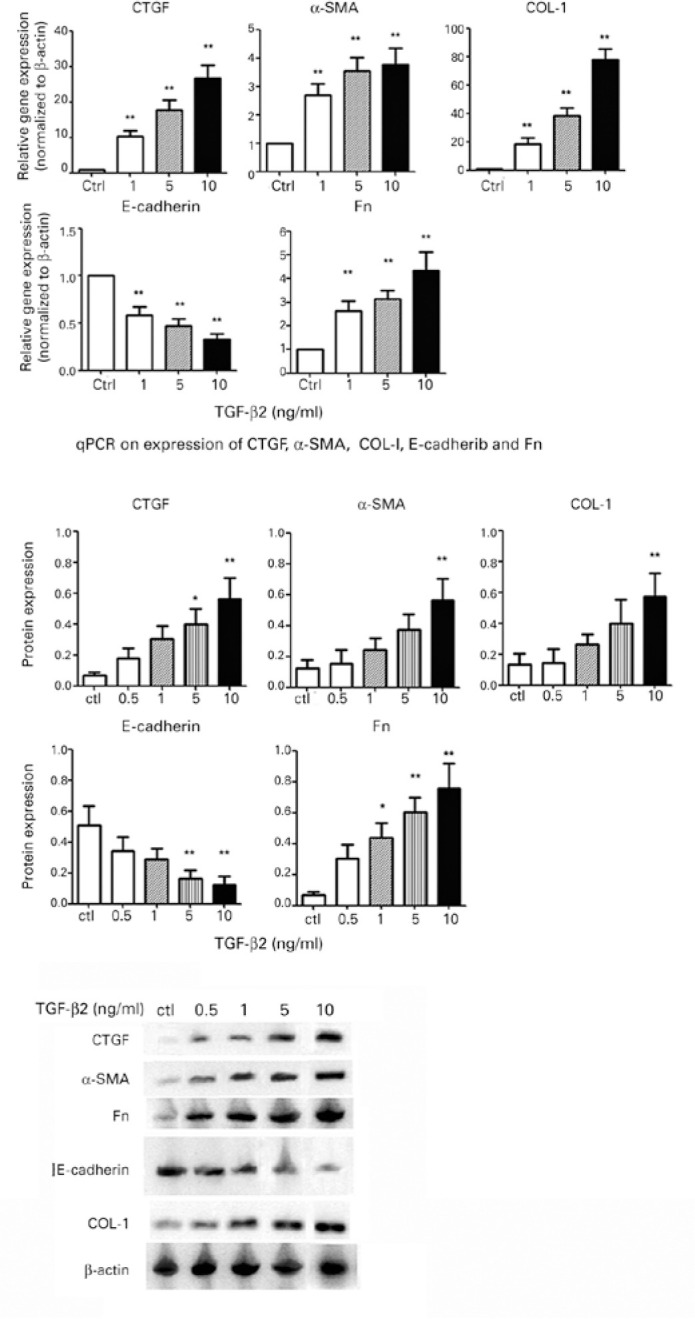
Expression of CTGF, _α_-SMA, COL-I, E-cadherin, and Fn: FHL 124 cells were treated with different concentrations of TGF-_β_2 (0-10 ng/mL) for 24h, and the expression of mRNA relevant to ACTB was detected via qPCR. The expression of protein relevant to ACTB was detected via Western blot analysis. P-values were provided as per respective “0” concentration: *p<0.05; **p<0.01

### Effect of AKF-PD on the mRNA and protein expression of TGF-_β_2, SMAD3, and SMAD4 in lens cells (Figure 3)

Our experiment showed that, after the 24-h treatment of lens cells with AKF-PD, the mRNA and protein levels of TGF-β2, SMAD3, and SMAD4 were dose dependently downregulated. The mRNA and protein levels of TGF-β2, SMAD3, and SMAD4 were significantly inhibited at 0.4 mg/mL (*p<0.05, **p<0.01) compared with the control group. However, the inhibitory effects on TGF-β2 and SMAD4 by Western blot analysis were not statistically significant in the 0.2 mg/mL AKF-PD groups (p>0.05).

**Figure 3:**
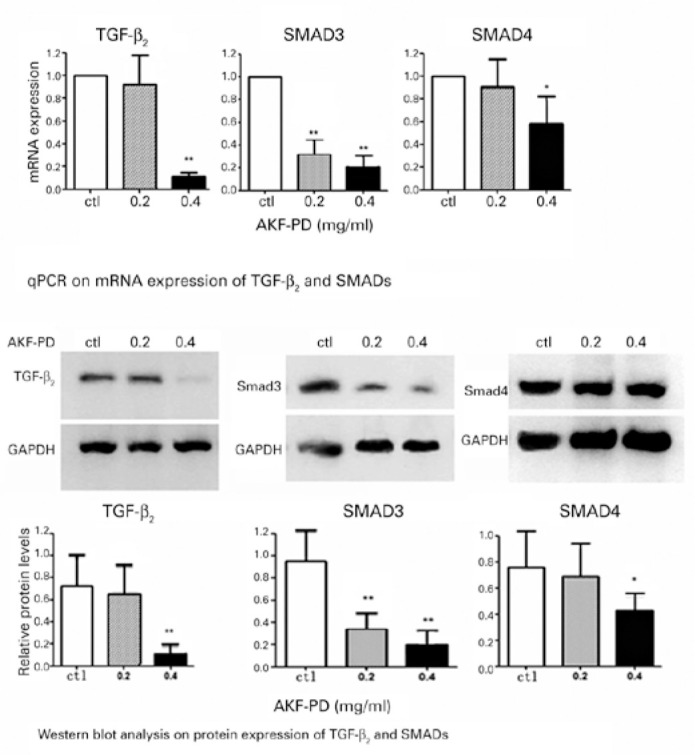
mRNA and protein expression of TGF-_β_2 and SMADs: The mRNA and protein expressions of TGF-_β_2 and SMADs were highly inhibited by 0.4 mg/mL AKF-PD (compared with the control group, *p<0.05; **p<0.01). The inhibitory effects on TGF-_β_2 and SMAD4 were not statistically significant in 0.2 mg/mL AKF-PD groups (p>0.05).

### Inhibitory effect of AKF-PD on TGF-_β_2 induced expression of SMAD mRNA and protein in lens cells (Figure 4)

Further experiments revealed that, in the presence of TGF-β2, AKF-PD also decreased the expression of the mRNA and protein levels of SMAD3 and SMAD4 dose dependently (*p<0.05; **p<0.01). However, the inhibitory effect on mRNA and protein expression of SMADs was detected but was not statistically significant in the 0.2 mg/mL AKF-PD groups (p>0.05).

**Figure 4:**
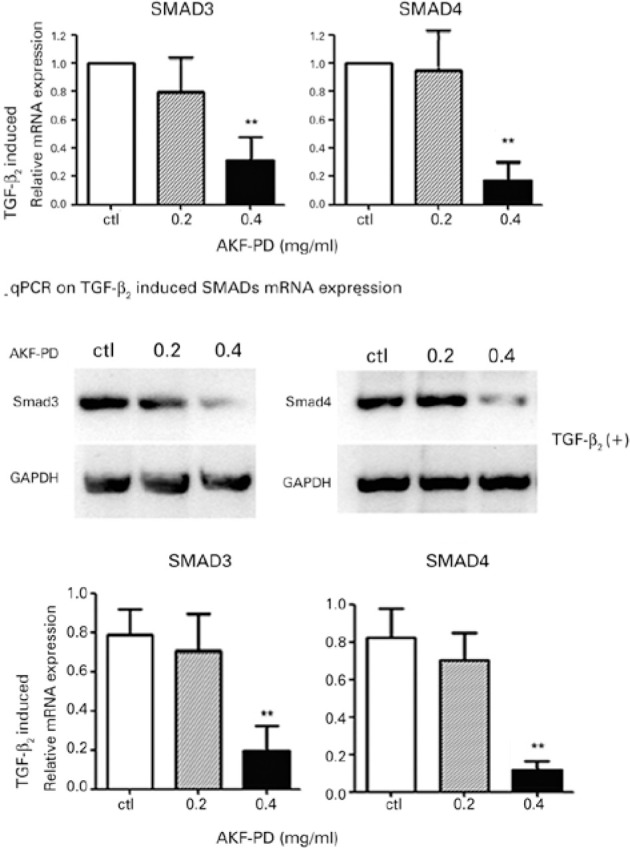
SMADs expression induced by TGF-_β_2: mRNA and protein expressions of SMADs induced by TGF-_β_2 were inhibited in 0.4 mg/mL AKF-PD groups (**p<0.01). The inhibitory effect on SMADs was detected but was not statistically significant in 0.2 mg/mL AKF-PD groups (p>0.05).

### Morphology observation of lens cells in vitro

**Figure 5:**
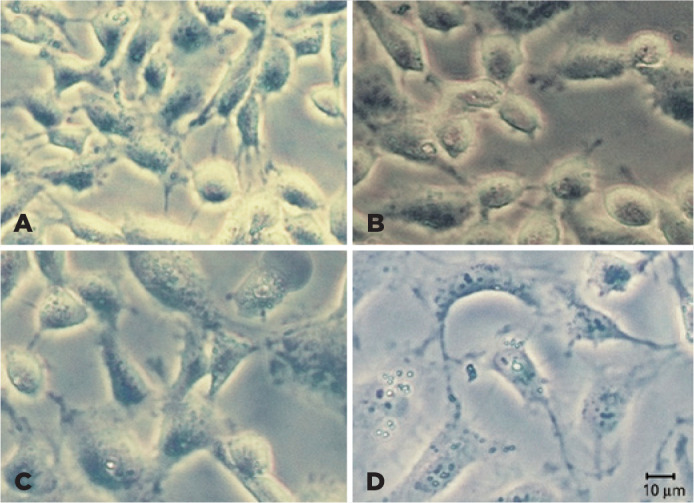
The three-dimensional images of FHL 124 cells in vitro: (A) Cells after treatment with 10 ng/mL TGF-_β_2 and 0.4 mg/mL AKF-PD. (B) Control group. (C) Cells after treatment with AKF-PD 0.4 mg/mL for 24h. (D) Cells after treatment with 10 ng/mL TGF-_β_2.

There was no morphological change in lens cells treated with 0.4 mg/mL AKF-PD (C) compared with control cells (B) after 24 h. Cells were spindled, starred, elongated, or irregular in shape after treatment with 10 ng/mL TGF-β2 (D), which were inhibited by 0.4 mg/mL AKF-PD (A) (Figure 5).

### Protein expression of COL-I, Fn, CTGF, and _α_-SMA was also analyzed by immunocytochemistry assay (Figure 6)

After treatment with 0.4 mg/mL AKF-PD for 24h, the protein expression of COL-I, Fn, CTGF, and α-SMA was significantly decreased (*p<0.05). The brown stains were highly densified by TGF-β2; however, they were faded by AKF-PD (*p<0.05).

**Figure 6:**
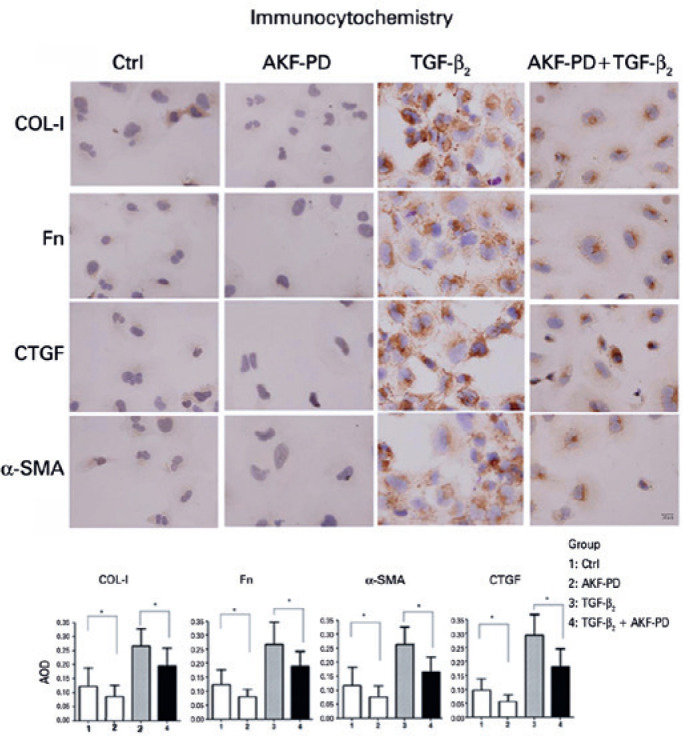
Expression of CTGF, _α_-SMA, COL-I, and Fn in FHL 124 cells analyzed by immunocytochemistry assay: AKF-PD inhibited CTGF, _α_-SMA, COL-I, and Fn protein expression (*p<0.05). TGF-_β_2 enhanced all protein expressions. However, the effect were inhibited by 0.4 mg/mL AKF-PD (*p<0.05).

## DISCUSSION

Previous studies have proven that AKF-PD functions as an antifibrotic agent in the pulmonary and renal fibrosis models^(16)^. It also ameliorates the progression of pulmonary hypertension induced by hypoxia in rats through its regulation of TGF-β expression and synthesis of ECM^(16)^. Besides targeting TGF-β-SMADs signal pathway, AKF-PD attenuates inflammation by inhibiting the NF-кB pathway in human proximal tubule cells^(17)^. Furthermore, through blockage of the Fas/Fas L pathway^(18)^, AKF-PD inhibits AngII-induced apoptosis of renal tubular cells, which can be initiated by the binding of lethal ligands, such as FAS/CD95 ligand, tumor necrosis factor (TNF)-α, and TNF (ligand) superfamily member 10 (best known as TNF-related apoptosis-inducing ligand), to various death receptors (i.e., FAS/CD95, TNF-α receptor 1, and TNF-related apoptosis-inducing ligand receptors 1 and 2, respectively)^(19)^. AKF-PD is a newly developed drug with antifibrotic activities through inhibiting various signal pathways and sheds new light on treatments of progressive fibrotic diseases^(20)^.

AKF-PD shows inhibitory effects through SMAD signal pathways^(20)^. The function of TGF-β in promoting fibrosis is principally mediated by the SMAD signaling pathway^(21,22)^. As an activator, TGF-β unites with TβR-II (one of the TGF-β ligands) to form the TβR-II- TGF-β-TβR-I tripolymer. The binding of TGF-β with its receptor II (TβRII) activates the kinase of TGF-β receptor I (TβRI). TβRI is phosphorylated and then phosphorylates SMAD2 and SMAD3. Subsequently, phosphorylated SMAD2 and SMAD3 bind to SMAD4 to constitute a SMAD complex. Then, the complex is shifted to the nucleus to regulate the transcription of target genes^(23)^. Finally, downstream biological effects were activated by SMAD pathway, where TGF-β signaling is activated and fibrosis of related tissue is enhanced by CTGF^(24)^. Moreover, there were increased expressions of CTGF-mRNA accompanied with stronger synthesis of collagen I and α-SMA in the residual debris of PCO^(25)^. Furthermore, α-SMA is an important sign of EMT and ECM synthesis in HLECs. Moreover, both α-SMA and E-cadherin, which are involved in EMT in HLECs^(26)^, help mediate cell-matrix adherence and myofibroblast^(27)^.

There are other signaling pathways, including ERK1/2, p38 MAPK, JNK, STAT3, and PKC. These pathways are also involved in the TGF-β-induced upregulation of CTGF expression in other cell types^(28)^. Many other transcription factors and microRNAs also regulate CTGF expression^(29)^. CTGF can promote cell mitosis and proliferation of fibroblasts and synthesize collagen, mediate cell adhesion, enhance fibrosis, and regulate ECM synthesis^(30)^.

In this study, the authors initially demonstrated an inhibitory effect of AKF-PD on TGF-β2-induced proliferation and EMT of HLEC line FHL 124. The effect acted in a dose-dependent manner. The authors found that cell proliferation was significantly suppressed at 0.6 mg/mL AKF-PD, and inhibition reached its climax at 1.0 mg/mL. Moreover, LDH assay indicated that there were no significant toxic effects at the concentrations of 0, 0.2, and 0.4 mg/mL. This result showed a hopeful prospect that AKF-PD may become a new therapeutic drug for PCO. The authors provided new evidence that TGF-β2 increased the expression of CTGF, α-SMA, COL-I, and Fn but decreased the expression of E-cadherin in the cell line. In contrast, AKF-PD showed its inhibitory effect by depressing TGF-β2-SMAD signaling pathway: AKF-PD suppressed expression of SMADs, CTGF, α-SMA, COL-I, and Fn, in the absence or presence of TGF-β2 stimulation, dose dependently.

Although AKF-PD showed remarkable suppression on proliferation and EMT of HLECs, there are still other issues that need to be managed. Cytotoxicity to corneal endothelial cell needs to be further detected. Due to non-regeneration of these cells, drugs that interfere with cell metabolism must be safe and nontoxic. The metabolism of RPE cells after AKF-PD treatment needs to be elucidated through the following studies. RPE is an important barrier for the retinal vessel and nerve cells. The RPE layer provides a stable microenvironment that prevents the leakage from choroid vessel and is extremely vital for normal retinal metabolism. In vivo studies are also urgent for the whole AKF-PD experimental series. Since lens cells are exposed to the aqueous humor in vivo, whether the liquid environment influences the effects of AKF-PD on lens must be investigated in the following steps. In the future use of eyedrops, nanoparticles could be applied to prolong and enhance the drug retention^(31)^. Nanoparticles are ideal and successful control release carriers for many medications^(31)^. An in vivo study on rabbit eyes confirmed that chitosan-sodium alginate nanoparticles could increase the 5-FU level in the aqueous humor compared to the native 5-FU solution^(32)^. Whether nanoparticles-AKF-PD can effectively inhibit HLEC EMT in vivo and how it influences corneal endothelium need to be explored in future experiments.

Therefore, AKF-PD exhibited powerful inhibitory effect on EMT of FHL 124 cells in the absence or presence of TGF-β2 stimulation in vitro. In addition, the inhibition on lens cells was not mediated by cytotoxicity. It may be a new strategy to inhibit EMT and prevent or treat PCO. Since laser capsulectomy is unavailable for infants, this strategy provides a promising therapy for infant PCO.
